# Comparative performance of ICOPE Step 1 and fried frailty criteria in detecting frailty phenotypes: A cross-sectional study

**DOI:** 10.1016/j.jarlif.2025.100036

**Published:** 2025-12-31

**Authors:** Clément Rimlawi, Marine Dexet, Abdoul R. Sawadogo, Gilles Kehoua, Myriam Le Goff, Olivier Villeneuve, Muriel Grau, Caroline Gayot, Achille Tchalla

**Affiliations:** a Laboratoire VieSanté - UR 24134 (Vieillissement, Fragilité, Prévention, e-Santé), Institut OMEGA HEALTH, Université de Limoges, Limoges, France; b CHU de Limoges, Pôle HU Gérontologie Clinique, F-87042 Limoges, 2 Avenue Martin-Luther King, France; c Unité de Recherche Clinique et d'Innovation (URCI) en Gérontologie, CHU de Limoges, Pôle HU Gérontologie Clinique, Limoges, France; d IMT Atlantique, LATIM (INSERM 1101), Brest, France

**Keywords:** Frailty, Older people, ICOPE screening tool, Diagnostic performance

## Abstract

**Background:**

Frailty represents a significant public health challenge among aging populations. Early and accurate detection is vital for implementing timely interventions that may delay or prevent functional deterioration. Among the available assessment tools, The Fried frailty phenotype is widely recognized as a reference framework for assessing frailty. In parallel, the WHO’s ICOPE Step 1 has been developed as a tool to detect potential declines in intrinsic capacity. Considering its design and purpose, ICOPE Step 1 may be regarded as a feasible option for use as a screening tool in clinical and community settings; however, direct comparative analyses within the same population remain limited. This study aimed to evaluate the concordance between the ICOPE Step 1 tool and Fried criteria to inform and enhance frailty screening practices in both clinical and community-based settings.

**Methods:**

This cross-sectional study included 202 community-dwelling older adults aged ≥60 years (mean age 85.0 ± 4.5; 160 [79.2 %] females), categorized as non-frail, pre-frail, or frail based on Fried’s frailty phenotype and the WHO ICOPE Step 1 screening tool. The diagnostic performance of the ICOPE tool was assessed in comparison to Fried’s criteria by calculating sensitivity, specificity, and the area under the receiver operating characteristic (ROC) curve.

**Results:**

Compared to the reference Fried criteria, the ICOPE Step 1 tool identified a higher proportion of individuals as frail (63 % vs. 29 %) and fewer as robust (2 % vs. 18 %). Diagnostic performance analysis showed a sensitivity of 83.9 % and a specificity of 43.8 %, with an area under the ROC curve (AUC) of 0.639, indicating moderate discriminative ability.

**Conclusion:**

ICOPE Step 1 demonstrated high sensitivity as a rapid, community-based screening tool for identifying older adults at risk of frailty. While it cannot replace the diagnostic utility of the Fried phenotype due to its limited specificity, it serves as a valuable first-line instrument to guide further comprehensive geriatric assessment, particularly via ICOPE Step 2.

**Abbreviations**
ICOPE: Integrated Care for Older People
TP: True positive
TN: True negative
FN: False negative
FP: False positive
AUC: Area under the curve
ROC: Receiver Operating Characteristic

## 1 Introduction

Frailty is one of the most critical dimensions associated with aging. It is estimated that approximately 15 % of individuals over the age of 65 are considered frail in community-dwelling [1]. Clinically recognized as a geriatric syndrome, frailty reflects a state of increased vulnerability resulting from age-related declines in physiological function and in both physical and cognitive reserves [2]. This condition compromises an individual’s ability to cope with external stressors, thereby elevating the risk of adverse health outcomes, including loss of independence [3]. Frailty typically presents clinical features such as unintentional weight loss, diminished muscle strength, fatigue, slow gait speed, and reduced levels of physical activity. According to these criteria, individuals are classified as robust, prefrail or frail [4]. Current estimates suggest that between one-quarter and one-half of individuals aged 85 and older are frail, placing them at markedly increased risk for falls, hospitalization, pathologic cognition and mortality [3,5].

Early detection of frailty represents a critical window for intervention, as frailty is increasingly recognized as a dynamic and partially reversible condition [6]. Identifying individuals in the pre-frail stage is particularly important, as this intermediate state is associated with a significantly elevated risk of progression to full frailty [7,8]. Timely intervention at this stage has the potential to prevent or delay the onset of frailty, thereby helping to preserve functional independence and maintain quality of life [9].

While the Fried criteria remain the gold standard for assessing frailty in research settings, their implementation in clinical practice presents several limitations. The requirement for physical performance measurements, such as grip strength and gait speed, can be particularly challenging in certain clinical contexts, including intensive care units or among patients with cognitive impairments or mobility limitations [[10], [11], [12]].

The World Health Organization's ICOPE framework offers a person-centered, function-oriented approach to promoting healthy aging and preventing frailty by monitoring intrinsic capacity across five key domains: locomotion, cognition, psychological well-being, sensory capacity, and vitality [13]. The Step 1 screening tool enables early detection of functional decline through a simple, standardized assessment suitable for both clinical and self-administration. Implementation and validation studies have demonstrated its high feasibility, sensitivity, and reliability, as well as its utility in identifying older adults at increased risk for frailty, disability, and cognitive impairment [14].

Despite the growing recognition of both the Fried frailty phenotype and the WHO ICOPE Step 1 tool as valuable assessment instruments, few studies have directly compared their performance in identifying frailty profiles within the same population [15]. The Fried criteria focus narrowly on the physical components of frailty [16], whereas the ICOPE framework adopts a broader conceptualization, assessing intrinsic capacity across multiple domains, including cognitive, psychological, and sensory functions [17]. This fundamental difference in scope may lead to variation in the sensitivity and specificity of each tool in identifying vulnerable older adults.

Understanding the degree of concordance and divergence between these two approaches is crucial for informed clinical decision-making and public health planning. The ICOPE tool’s emphasis on early detection and its feasibility for self-administration make it particularly appealing for community-based screening initiatives [18]. In contrast, the Fried criteria remain a benchmark in research and clinical evaluation, supported by robust validation and strong predictive value for adverse health outcomes [19].

This study addresses a critical gap by systematically comparing the frailty profiles identified using Fried’s criteria with those detected through the ICOPE Step 1 screening tool. By analyzing the distribution of robust, pre-frail, and frail individuals across both assessment methods, the study aims to generate essential insights into the comparative performance of these tools in clinical practice.

## 2 Study methods

This cross-sectional study included a total of 202 participants aged between 60 and 99 years, recruited from various departments in France (Landes, Eure, and Haute-Vienne). Inclusion criteria required that participants be relatively healthy, free from acute illness, aged 60 years or older, and able to undergo complete assessment according to the Fried criteria as well as the WHO ICOPE step 1 screening protocol conducted by our research team. The study was conducted in accordance with the Declaration of Helsinki, and the study was approved by the ethical review board of Limoges University. Verbal non-opposition was obtained from all participants prior to data collection.

### 2.1 Frailty assessment

#### 2.1.1 Fried’s phenotype method

Frailty was classified via Fried's phenotype using five criteria: weight loss (>4.5 Kg/year), exhaustion, low activity (Minnesota Questionnaire: <383 kcal/week men/<270 women), slow walk (height-adjusted 15-ft times), and weak grip (BMI-stratified Jamar dynamometer). Participants were non-frail (0 criteria), pre-frail [[1], [2]], or frail (≥3) [16].

#### 2.1.2 ICOPE Step 1 method

This study implemented the WHO ICOPE guidelines [20], employing a nine-item screening tool in Step 1 to detect potential impairments across six intrinsic capacity domains: cognition, mobility, nutrition, vision, hearing, and mood. A decline in any domain was considered an impairment. A composite score ranging from 0 to 6 was constructed, with each domain impairment assigned one point. For instance, impairments in both vision and hearing yielded a cumulative score of 2 out of 6. Participants were classified according to the following criteria: a score of 0 indicated non-frail status, scores of 1 to 2 indicated pre-frail status, and scores ≥3 indicated frailty.

### 2.2 Statistical analysis

To assess the prevalence of frailty status among participants, descriptive frequency analyses were conducted, categorizing individuals as frail, pre-frail, or non-frail. Simultaneously, the diagnostic accuracy of the ICOPE Step 1 screening tool, designed to evaluate frailty through a multidimensional framework, was examined in relation to physical frailty as defined by the Fried phenotype model. The agreement between classifications from the ICOPE Step 1 tool and the Fried criteria was assessed using a contingency table, accompanied by a bar chart to illustrate potential classification discrepancies. For comparative analysis, both the ICOPE and Fried frailty variables were dichotomized by combining robust and pre-frail categories into a single “non-frail” group. Diagnostic performance metrics, including sensitivity (the proportion of correctly identified frail individuals), specificity (the proportion of correctly identified non-frail individuals), and the area under the receiver operating characteristic (ROC) curve (AUC) [21,22], were computed to evaluate the discriminatory capability of the ICOPE tool relative to the Fried criteria. The ROC curve was plotted to provide a graphical representation of the ICOPE tool’s performance in differentiating frail from non-frail participants. All statistical analyses were completed with JASP (GNU Affero General Public License).

## 3 Results

We observed a majority of women (79.21 %) and a predominance of employees and manual workers (66.34 %), suggesting a population generally from lower socioeconomic background (see Table 1). In addition, more than half of the participants had a primary education level (57.92 %). Most lived alone (64.85 %) and received support from a caregiver (81.19 %), reflecting a certain degree of social vulnerability. The median age of participants was 85 years, indicating a relatively elderly population. Regarding frailty profiles, more than half of the participants were in a state of pre-frailty (53.47 %), while only 18.81 % were considered robust according to Fried’s criteria. Table 2 shows the six altered intrinsic capacity domains according to ICOPE assessment. Most participants had an impairment in cognition (69.80 %) and vision (76.23 %), while approximately half presented with a locomotion impairment.

**Table 1:** Sociodemographic characteristics and Fried criteria of the cohort.

Variables	Population total (*N* = 202)
Age (median [Q1-Q3])	85 [79–88]
Female n (%)	160 (79.21)
Highest level of education Elementary school, n (%) High school, n (%) Post-secondary (college or university), n (%)	117 (57.92) 53 (26.24) 32 (15.84)
Socio-occupational category Employees and manual workers	134 (66.34)
Presence of a caregiver, yes n (%)	164 (81.19)
Living alone (n %)	131 (64.85)
Fried Criteria Robust Pre-frail Frail	38 (18.81) 108 (53.47) 56 (27.72)

**Table 2:** Domains alerts from ICOPE Step 1.

Intrinsic Domains	*N* = 202
Cognition n (%)	141 (69.80)
Nutrition n (%)	72 (35.64)
Vision n (%)	154 (76.23)
Hearing n (%)	55 (27.22)
Psychological n (%)	82 (40.59)
Locomotion n (%)	102 (50.49)

**Fig. 1:**
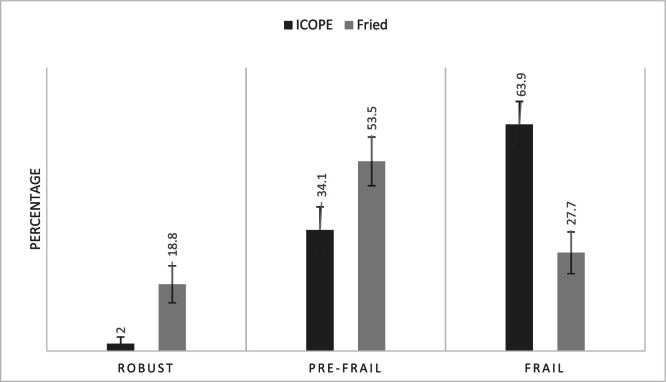
Frailty profiles.

**Fig. 2:**
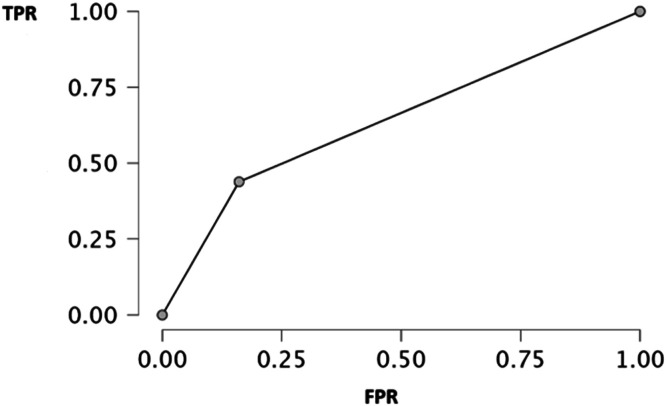
ROC analysis of the ICOPE Step 1 tool compared to the Fried phenotype for frailty detection. TPR true positive rate, FPR false positive rate.

To evaluate the ability of the ICOPE Step 1 tool to identify frailty, a comparison was conducted with the reference Fried test. Fig. 1 illustrates the distribution of individuals according to frailty profiles (robust, pre-frail, frail) obtained using both tools. We observed that among individuals classified as robust, only 2 % were designated as such by ICOPE compared to 18 % by Fried. Regarding frail subjects, 63 % were classified as frail by ICOPE, versus only 29 % with Fried. These data suggest that the ICOPE test tends to classify a larger number of individuals as frail compared to the Fried test. This trend was confirmed by the contingency Table 3. Among 202 participants, the ICOPE test identified 47 true positives (frail according to both tools), 9 false negatives (frail per Fried criteria but not identified by ICOPE), 82 false positives (classified as frail by ICOPE but non-frail per Fried), and 64 true negatives (not-frail to both tools). From these data, several performance indicators presented in Table 4 were calculated. We observed that sensitivity, reflecting the ability of ICOPE Step 1 to correctly identify frail individuals, was relatively high at 83.9 %. In contrast, specificity, indicating the capacity to correctly detect non-frail individuals, was lower at 43.8 %. The area under the curve (AUC) was 0.639, indicating moderate overall performance of the tool (a result is considered discriminatory when exceeding 0.7). Finally, Fig. 2 below, presenting the ROC (Receiver Operating Characteristic) curve, confirms these observations. The curve remains close to the diagonal, indicating modest performance in discriminating between frail and non-frail individuals relative to the Fried model.

**Table 3:** Contingency table comparing ICOPE Step 1 and fried test for frailty diagnosis.

Reference test	Index test		Total
	Frail	No-frail	
Frail	47 (TP)	9 (FN)	56
No-frail	82 (FP)	64 (TN)	146
Total	129	73	202

TP (True Positive), FP (False Positive), FN (False Negative), TN (True Negative).

**Table 4:** Performance indicators of the ICOPE Step 1 tool.

Indicators	Value (%)
Sensibility	83.9
Specificity	43.8
AUC	0.639

AUC area under the curve, * The Fried test was selected as the reference standard.

## 4 Discussion

The present findings highlight that the multidimensional ICOPE Step 1 tool showed good sensitivity for frailty detection, yet lower specificity, suggesting a propensity to overclassify individuals as frail relative to the exclusively physical Fried criteria. With an area under the ROC curve (AUC) of 0.639, its overall discriminative performance is considered moderate for distinguishing between different frailty profiles. The comparison between the ICOPE Step 1 screening tool and the Fried frailty model reveals distinct operational characteristics and clinical applications. ICOPE Step 1 demonstrates high sensitivity, effectively identifying individuals classified as frail by the Fried criteria. However, its lower specificity indicates a tendency to flag individuals as frail who are deemed non-frail under the Fried model. This divergence stems from ICOPE's multidimensional framework, which evaluates not only physical domains but also cognitive, nutritional, sensory, and psychological dimensions of intrinsic capacity [18].

Consequently, ICOPE does not supplant the Fried model, which is a more specific instrument centered on physical frailty [23]. Instead, it complements it by enabling broader and earlier detection of vulnerabilities. This justifies ICOPE's deployment in large-scale screening contexts, where its capacity to capture early functional decline signals is evidenced by the low proportion of individuals classified as robust by ICOPE, even among those considered robust by Fried criteria. Nevertheless, ICOPE Step 1 alone cannot definitively exclude frailty; it necessitates confirmation through Step 2′s comprehensive assessment to refine evaluations and guide personalized interventions. A study confirmed that the ICOPE tool demonstrates high sensitivity (94.6 %) in detecting intrinsic capacity decline, supporting its reliability as an initial screening instrument in community settings [24].

## 5 Conclusion

In summary, ICOPE Step 1 represents an effective preliminary screening instrument that triages individuals for comprehensive evaluation when indicated, in line with proactive strategies aimed at preventing functional decline in older age. Its deployment does not replace the diagnostic role of the Fried frailty phenotype; instead, it enhances holistic geriatric assessment by encompassing a broader range of vulnerabilities.

This study has several notable limitations. First, while ROC was used to compare two frailty tools, it primarily reflects discrimination and does not capture predictive value, calibration, or direct clinical utility [22,25]. ROC curves are also insensitive to prevalence and do not account for the unequal consequences of misclassification, such as the higher risk associated with missing frail individuals [26].Moreover, our relatively small sample size may limit the stability of AUC estimates [21]and restrict the generalizability of our findings. The limited availability of comparable studies further complicates the contextualization of our results within the existing literature. Finally, the absence of a standardized, validated methodology for classifying older adults as robust, pre-frail, or frail introduces a degree of subjectivity to our approach, underscoring the need for more robust reference standards in future research.

Crucially, ICOPE Step 1 is designed purely as a screening tool, whereas the Fried phenotype serves diagnostic purposes. This distinction necessitates progression to ICOPE Step 2 for a more comprehensive assessment of multidomain frailty.

Nonetheless, our data confirm a high sensitivity for detecting intrinsic capacity decline using ICOPE Step 1, demonstrating its reliability as an effective, rapid, and practical community-based screening method. Despite this, its limited specificity means it cannot definitively diagnose frailty, reinforcing the indispensable role of Step 2 in identifying multidomain impairments.

ICOPE Step 1 remains a valuable frontline tool in public health for early detection. It efficiently identifies individuals who warrant deeper geriatric evaluation, ensuring timely and targeted interventions.

## Availability of data and materials

The datasets analyzed in the current study are available from the corresponding author upon request.

## Ethics approval and consent to participate

We confirm that all methods were performed in accordance with the Declaration of Helsinki and relevant regulations and guidelines. This study has been registered with the French Health Data Hub: N°F20220214102025. Oral non-opposition was obtained from all participants**.**

## CRediT authorship contribution statement

**Clément Rimlawi:** Writing – review & editing, Writing – original draft, Visualization, Validation, Methodology, Investigation, Formal analysis, Data curation, Conceptualization. **Marine Dexet:** Writing – review & editing, Methodology, Investigation, Formal analysis, Data curation. **Abdoul R. Sawadogo:** Validation, Investigation, Formal analysis. **Gilles Kehoua:** Validation, Investigation, Formal analysis. **Myriam Le Goff:** Writing – review & editing. **Olivier Villeneuve:** Investigation. **Muriel Grau:** Investigation. **Caroline Gayot:** Writing – review & editing, Conceptualization. **Achille Tchalla:** Writing – review & editing, Supervision, Methodology, Conceptualization.

## Declaration of competing interest

The authors declare that they have no known competing financial interests or personal relationships that could have appeared to influence the work reported in this paper.

**Acknowledgements**

Declaration of the use of AI-assisted technologies to improve the grammatical accuracy of this article.
